# Molybdenum Foliar Fertilization Improves Photosynthetic Metabolism and Grain Yields of Field-Grown Soybean and Maize

**DOI:** 10.3389/fpls.2022.887682

**Published:** 2022-05-25

**Authors:** Sirlene Lopes Oliveira, Carlos Alexandre Costa Crusciol, Vitor Alves Rodrigues, Tatiani Mayara Galeriani, José Roberto Portugal, João William Bossolani, Luiz Gustavo Moretti, Juliano Carlos Calonego, Heitor Cantarella

**Affiliations:** ^1^Department of Crop Science, College of Agricultural Sciences, São Paulo State University, Botucatu, Brazil; ^2^Soils and Environmental Resources Center, Agronomic Institute of Campinas (IAC), Campinas, Brazil

**Keywords:** *Glycine max* L. (Merr.), *Zea mays* L., stimulant effect, photosynthetic activity, nitrogen metabolism, carbon metabolism

## Abstract

Foliar fertilization has been used as a supplemental strategy to plant nutrition especially in crops with high yield potential. Applying nutrients in small doses stimulates photosynthesis and increases yield performance. The aim of this study was to evaluate the efficiency of foliar application of molybdenum (Mo) to soybean and maize. The treatments consisted of the presence (+Mo) and absence (-Mo) of supplementation. Plant nutritional status, nitrate reductase (NR) activity, gas exchange parameters, photosynthetic enzyme activity (Rubisco in soybean and maize and PEPcase in maize), total soluble sugar concentration, leaf protein content, shoot dry matter, shoot nitrogen accumulated, number of grains per plant, mass of 100 grains, and grain yield were evaluated. For soybean and maize, application of Mo increased leaf NR activity, nitrogen and protein content, Rubisco activity, net photosynthesis, and grain yield. These results indicate that foliar fertilization with Mo can efficiently enhance nitrogen metabolism and the plant’s response to carbon fixation, resulting in improved crop yields.

**Graphical Abstract:**
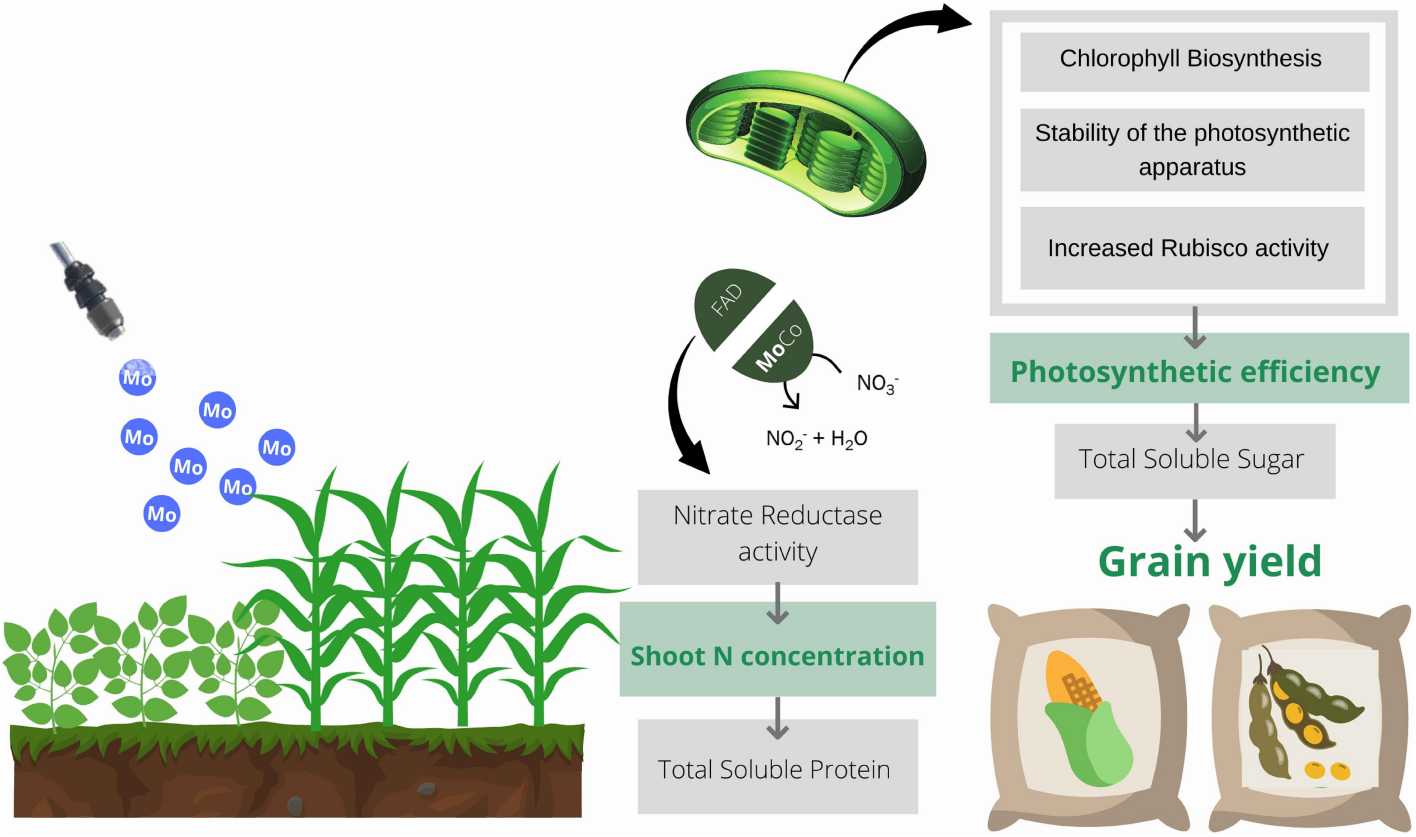
Schematic representation of the role of molybdenum in plants. Molybdenum acts in the assimilation of N in plants through the nitrate reductase enzyme. Mo also acts directly on the photochemical phase of photosynthesis through chlorophyll biosynthesis and stability of the photosynthetic apparatus. Increased photosynthetic activity activates nitrate reductase and promotes the initiation of proteins translation. Thus, there is an increase in the content of total soluble protein in the leaves, which may lead to an increase in the content and activity of the rubisco enzyme. This enzyme is responsible for carbon fixation during the production of carbohydrates, leading to an increase in the content of total soluble sugars in plants, culminating in greater grain yield.

## Introduction

Soybean [*Glycine max* L. (Merr.)] and maize (*Zea mays* L.) are some of the most important crops worldwide (Xu et al., 2020), with global areas of approximately 120 and 197 million hectares, respectively, in 2019 (Food and Agriculture Organization of the United Nations, 2019). The increasing demand for food has heightened interest in technologies for optimizing agricultural production without increasing the consumption of natural resources (Schröder et al., 2019). One such strategy is foliar fertilization, which is an important technique for additional nutrient supply, increasing plant growth and production, even when plants are not nutrient deficient (Rosolem, 2002). Because of the small amounts of Mo required by most plants, Mo application with solid fertilizers at seeding is not always done; therefore, the supply of Mo to field crops may be neglected. The application of small doses of nutrients strategically at specific phenological stages can stimulate carbon metabolism and enhance tolerance to abiotic stresses such as drought stress (Primavesi, 1978; Alexander, 1986; Hussain et al., 2021; Rodrigues et al., 2021). However, gaps remain in the understanding of the effects of supplemental fertilization with micronutrients, especially molybdenum (Mo), on plant metabolism and production under field conditions.

Mo is a component of enzymes that are essential for the absorption, assimilation and transport of nitrogen (N) in plants (Mendel, 2013; Bittner, 2014). Consequently, Mo deficiency or supplementation indirectly affects the products of N metabolism (Marschner, 2012). Mo is typically applied with the aim of stimulating biological nitrogen fixation (BNF) and nitrate reduction (Calonego et al., 2010; Mercante et al., 2011; Crusciol et al., 2019). Under Brazilian field conditions, the application of Mo *via* seed or leaves has been shown to be efficient in increasing nodulation, nitrate reductase activity, protein content, leaf area, number of pods, and grain yield (Calonego et al., 2010; Almeida et al., 2013; Silva et al., 2017; Crusciol et al., 2019); however, recent research has demonstrated that Mo also directly impacts photosynthesis due to its involvement in chlorophyll biosynthesis and stability of the photosynthetic apparatus (Yu et al., 2006; Imran et al., 2019). In addition, enhancing photosynthesis by adding Mo increases water use efficiency (WUE), leading to greater tolerance of abiotic stresses (Wu et al., 2020). However, these results were obtained in experiments performed under controlled conditions, and the actual effects of Mo supplementation on crop physiology and productivity under field conditions remain unclear. We hypothesized that Mo stimulates N and carbon metabolism to increase photosynthesis and plant productivity. To test this hypothesis, we investigated the effect of foliar application of Mo on the physiology and grain yield of soybean and maize.

## Materials and Methods

### Description of the Experimental Area

The field experiments consisted of two soybean crops in the summer 2018/2019 and 2019/2020 and two maize crops in the autumn/winter 2019 and 2020 (second season maize). All crops were grown in a rainfed cropping system. The experiments were conducted at the Experimental Lageado farm of São Paulo State University (UNESP) in the southeastern region of São Paulo State, Brazil (48° 26′ West, 22° 51′ South, and elevation 786 m above sea level). The soil is classified as a Ferralsol, which corresponds to the clayey textural class, kaolinitic, thermic Typic Haplorthox (United States Department of Agriculture, 2014). The area where the experiment was conducted had remained under no-till management for 12 years, in which soybean has been cultivated. According to the Köppen classification, the prevailing climate in the region is warm-moderate (mesothermal, with rainy summers and dry winters), corresponding to type Cwa. The average annual rainfall is 1,360 mm, and the mean annual air temperature is 20.7°C (50-year average; UNICAMP, 2020).

**Figure 1:**
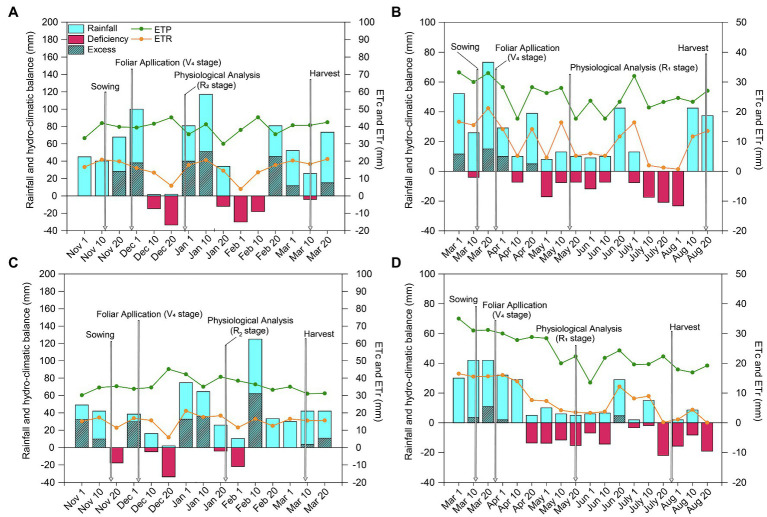
Climatological hydroclimatic balance for soybean (**A**, 2018/19; **C**, 2019/20) and maize (**B**, 2019; **D**, 2020) crops grown in Botucatu, SP, Brazil. ETc: crop evapotranspiration; ETr: real evapotranspiration. Arrows indicate the timing of spraying and sampling. Different lowercase letters represent statistical difference between treatments (presence or absence of Mo) according to the F test (*p* ≤ 0.10).

To monitor the water balance (Figure 1), the water retention capacity of the soil was obtained from a Richards extraction chamber stress table (Cassel and Nielsen, 2018) to determine the soil water potential (ψw). During the experimental period, data on rainfall, maximum and minimum air temperatures, and evapotranspiration (ET_0_) were collected from a meteorological station located near the experimental plot. Climatological water balances were determined according to the method of Rolim et al. (1998) following the procedure of Thornthwaite and Mather (1995).

Soil samples were obtained from a depth of 0.00–0.20 m to evaluate texture (Donagemma et al., 2017) and chemical properties (Van Raij et al., 2001), which are shown in Supplementary Table 1.

### Experimental Design and Treatments

The treatments were characterized by the presence (+Mo) and absence (-Mo) of Mo in a randomized block design with 12 replicates. Foliar spraying was performed at the V_4_ phenological stage of soybean and maize in both growing seasons (Fehr and Caviness, 1977; Ritchie et al., 1993). This phenological stage is critical for the definition of the productive potential of maize and the nodulation in soybean intensifies, also coinciding with the increase in the absorption of nutrients in both crops, such as nitrogen (Hanway, 1966; Campo et al., 1999; Fancelli and Dourado Neto, 2000; Bender et al., 2015). Spraying was carried out with the application of 180 L ha^−1^ of a water solution containing potassium molybdate [30 g Mo ha^−1^, (K_2_MoO_4_)] and an organosilicon adjuvant at a dose of 30 ml ha^−1^ (polydimethylsiloxane, d = 1.1 g cm^−3^). This dose of Mo was based on several studies in legumes and cereals whose best responses were obtained with doses close to 30 g Mo ha^−1^ (Vieira et al., 1998; Albino and Campo, 2001; Zoz et al., 2012). An aerograph atomizer propelled by CO_2_ with a working pressure of 1.8 bar was used for foliar spraying. The boom included six fat flan nozzles (TTI 110 02 VP) spaced 0.5 m apart operating at a distance of 0.5 m from the crop canopy and a velocity of 1 m s^−1^.

### Fertilization and Crop Management

#### Soybean

Soybean genotype TMG 7062 RR (Tropical Breeding & Genetics®) was used. Before sowing, seeds were treated with fungicides (100 g carboxin + 100 g a.i. thiram 100 kg^−1^ seeds) and inoculated with SEMIA 5079 (*Bradyrhizobium japonicum*) and SEMIA 5080 (*Bradyrhizobium diazoefficiens*). Each plot consisted of 10 rows with a length of 10 m and an inter-row spacing of 0.45 m, corresponding to an area of 45 m^2^. Sowing was carried out to obtain a population of 310,000 plants ha^−1^. Fertilizer at sowing was 300 kg ha^−1^ of 00-20-20 (60 kg ha^−1^ P_2_O_5_ and 60 kg ha^−1^ K_2_O) in both soybean growing seasons. Management for weeds, pests, and diseases was performed as recommended for the crop.

#### Maize

The maize crop was grown in succession to soybean. Maize seeds (P3707VYH Hybrid; Pioneer®) were treated with fungicides (carboxin + thiram, 1 g ai. kg^−1^) before sowing. Maize was sown in plots containing 10 rows with a length of 10 m and inter-row spacing of 0.45 m. The maize sowing density was 3.1 seeds meter^−1^ to give an estimated stand of 68,888 plants ha^−1^. Fertilization at sowing consisted of 300 kg ha^−1^ of the 08–28-16 formula (24 kg ha^−1^ of N, 84 kg ha^−1^ of P_2_O_5_ and 48 kg ha^−1^ of K_2_O) in both growing seasons. Fertilization with N and K was carried out at stage V_6_ by spreading 100 kg N ha^−1^ as ammonium sulfate and 20 kg K_2_O ha^−1^ as potassium chloride (Cantarella et al., 1997) over the soil surface, keeping a distance of approximately 2 cm from the maize crop. Pest and diseases control were carried out according to the cultivation recommendations.

### Nutritional, Physiological, and Biochemical Analyses

The nutritional and biochemical analyses of soybean and maize plants were carried out at stages R_2_ and R_1_, respectively, with the collection of 20 leaves (for each analysis) within each plot, considering the third fully developed leaf of the main stem for the crop of soybean (Ambrosano et al., 1997), and the middle third of the leaves below and opposite the ear for maize (Ritchie et al., 1993; Cantarella et al., 1997). At these same phenological stages, the photosynthetic parameters were analyzed, taking 10 plants from each plot, considering the median leaflet of the third fully developed leaf of the main stem of soybean and the medium third of 10 leaves of the ear of maize.

#### Analysis of Crop Nutritional Status

Leaf macro and micronutrient concentrations were determined from samples of 20 dried and ground leaves. To determine N content, samples were subjected to sulfur digestion followed by Kjeldahl distillation (Kirk, 1950). For the determination of other macronutrients (P, K, Ca, Mg, and S) and micronutrients (Mo, Fe, Mn, B, Cu, and Zn), the samples were subjected to nitroperchloric digestion and subsequent atomic absorption spectrophotometry according to the methodology described by Malavolta et al. (1997).

#### Total and Active Nitrate Reductase Determination

Nitrate reductase (NR) activity was determined according to the methodology described by Hewitt and Nicholas (1964). Fresh leaves were macerated in liquid nitrogen at a ratio of 1 g fresh leaves to 2 ml of extraction buffer (25 mM Tris–HCl (pH 8.5), 1 M EDTA, 1 mM DTT, 1% BSA, 20 μM FAD and leupetin 200 μM). After centrifugation (14,000 ×*g*; 10 min; 4°C), 200 μl of the supernatant was added to reaction buffer to obtain a final volume of 0.5 ml. The buffer solution consisted of Hepes-KOH, pH 7.6 (50 mM); 10 mM MgCl_2_ (for active NR) or 5 mM EDTA (for total NR); 10 μM FAD; 3% casein; and 1 mM DTT. The extract for determination of total NR was incubated for 10 min with 11 μl of a solution containing 250 mM AMP and 500 mM EDTA. To start the reaction, 25 μl of 5 mM NADH diluted in potassium phosphate buffer (100 mM KPO_4_), pH 7.0, was added to start the reaction. The reaction was incubated for 30 min and stopped *via* the addition of 62.5 μl of 500 mM zinc acetate. The solution was then centrifuged, and the supernatant was used for colorimetric determination of nitrite formation (Hageman and Reed, 1980).

#### Photosynthetic Parameters

Photosynthetic parameters were determined by non-destructive evaluation of leaves using a portable gas exchange analyzer (CIRAS-3 Portable Photosynthesis System, PP Systems Inc., Amesbury, MA, United States). The instrument measurement conditions were standardized to 380–400 mol^−1^ atmospheric CO_2_, 1,100 μmol quanta m^−2^ s^−1^ photosynthetically active radiation (PAR) delivered by LED lamps, a leaf chamber temperature of 25°C–27°C, and 60%–70% relative humidity. The minimum equilibration time for each set of measurements was 3 min. Measurements were conducted between 10:00 am. and 12:00 pm. The following attributes were evaluated: net photosynthetic rate (*A*; μmol CO_2_ m^−2^ s^−1^), stomatal conductance (*gs*; mol H_2_O m^−2^ s^−1^), internal CO_2_ concentration in the substomatal cavity (*Ci*; μmol mol^−1^), transpiration (*E*; mmol H_2_O mm^−2^ s^−1^), WUE (μmol CO_2_ (mmol H_2_O)^−1^) calculated by the *A/E* ratio and obtained through instant reading, and carboxylation efficiency obtained by the *A/Ci* ratio.

#### Total Leaf Protein Concentration

Total leaf protein concentration was measured according to the method proposed by Bradford (1976), which is based on the hypsochromic effect from 465 to 595 nm resulting from the selective binding of the dye Coomasie® Brilliant Blue G-250 to proteins containing basic and aromatic amino acids (Wenrich and Trumbo, 2012). Total protein content was determined by referring to a standard curve constructed from bovine serum albumin and expressed as mg g^−1^ fresh weight.

#### Total Soluble Sugar Concentration

Total soluble sugar concentration was determined by the sulfur-phenol method, in which simple sugars are dehydrated by sulfuric acid and complexed with phenol. The color change of the solution is measured in the visible range and is proportional to the content of total sugars in the sample (DuBois et al., 1956). The concentrations were determined by referring to a standard sucrose curve and expressed in g kg^−1^.

#### Photosynthetic Enzymes

The activity of phosphoenolpyruvate carboxylase (PEPcase) was measured in maize leaves using the enzymatic method coupled with the oxidation of NADH (Degl’Innocenti et al., 2002). Enzyme activity was measured by recording the decrease in absorbance at 340 nm over 300 s, and the results were expressed in μmol min^−1^ mg of protein^−1^.

Ribulose-1,5-bisphosphate carboxylase/oxygenase (Rubisco) activity was determined in soybean and maize using the same methodology as for PEPcase (Reid et al., 1997). Rubisco activity was calculated from the difference in absorbance readings at 0 and 1 min and expressed as μmol min^−1^ mg protein^−1^.

### Agronomic Parameters and Grain Yield

Upon physiological maturity of soybean and maize, plants were manually harvested from an area of 15 m^2^ within each plot, and the following variables were evaluated: number of pods per plant (soybean), prolificacy (maize), number of grains per plant (NGP), weight of 100 grains (W100G) expressed in g; and grain yield (GY), expressed in kg ha^−1^, and corrected for 13% moisture. Moisture content was determined using an automatic moisture meter (Gehaka G650i, Brazil). Five plants were harvested from the soil surface in the two central lines of each plot and subjected to 70°C to complete the drying process. The mass of dry plants was measured to obtain shoot dry matter (SDM) and then ground. Finely ground plant material was analyzed for nitrogen concentration in grains, stems and leaves, as described above, in order to measure shoot nitrogen concentration (SNC) and shoot nitrogen accumulated (SNA) in the plant.

### Data Analysis

Data were first tested for normality using the Shapiro–Wilk method (Shapiro and Wilk, 1965) and for homoscedasticity using Levene’s test (Levene, 1960), both with *p* ≤ 0.10. Foliar fertilization was considered a fixed factor, and year was considered a random factor. Subsequently, the means were subjected to one-way analysis of variance (ANOVA) using the F-test (*p* ≤ 0.10). We built a heatmap of the Pearson correlation coefficients (*p* ≤ 0.05) among the measured variables.

## Results

### Weather Conditions

Total rainfall was significantly higher during the 2018/19 soybean growing season (590 mm) than the 2019/20 soybean growing season (434 mm; Figures 1A,C). However, the rainfall distribution was better in the second season than the first, which was characterized by two water deficit events. The first occurred at the end of the vegetative stage (between phenological stages V_5_ and V_8_) and the beginning of the reproductive stage (R_1_), while the second occurred at full flowering (R_2_). While there were periods of water deficit at the beginning of the vegetative stage and in the reproductive stage (in R_2_ and at the end of R_4_) in the second growing season, these events were less intense than those in the first growing season. In both soybean growing seasons, there was a water deficit at stage V5. The occurrence of a water deficit at this stage is particularly important because the soybean crop intensifies water use, nodulation of BNF, and dry matter accumulation.

Compared with the soybean growing seasons, rainfall was significantly lower in both maize growing seasons (319 and 293 mm total precipitation during the first and second growing season, respectively; Figures 1B,D), resulting in long periods of water deficit that lasted almost the entire crop cycle and were greater and more intense in the second growing season.

### Nutritional Status and Nitrate Reductase Activity

**Figure 2:**
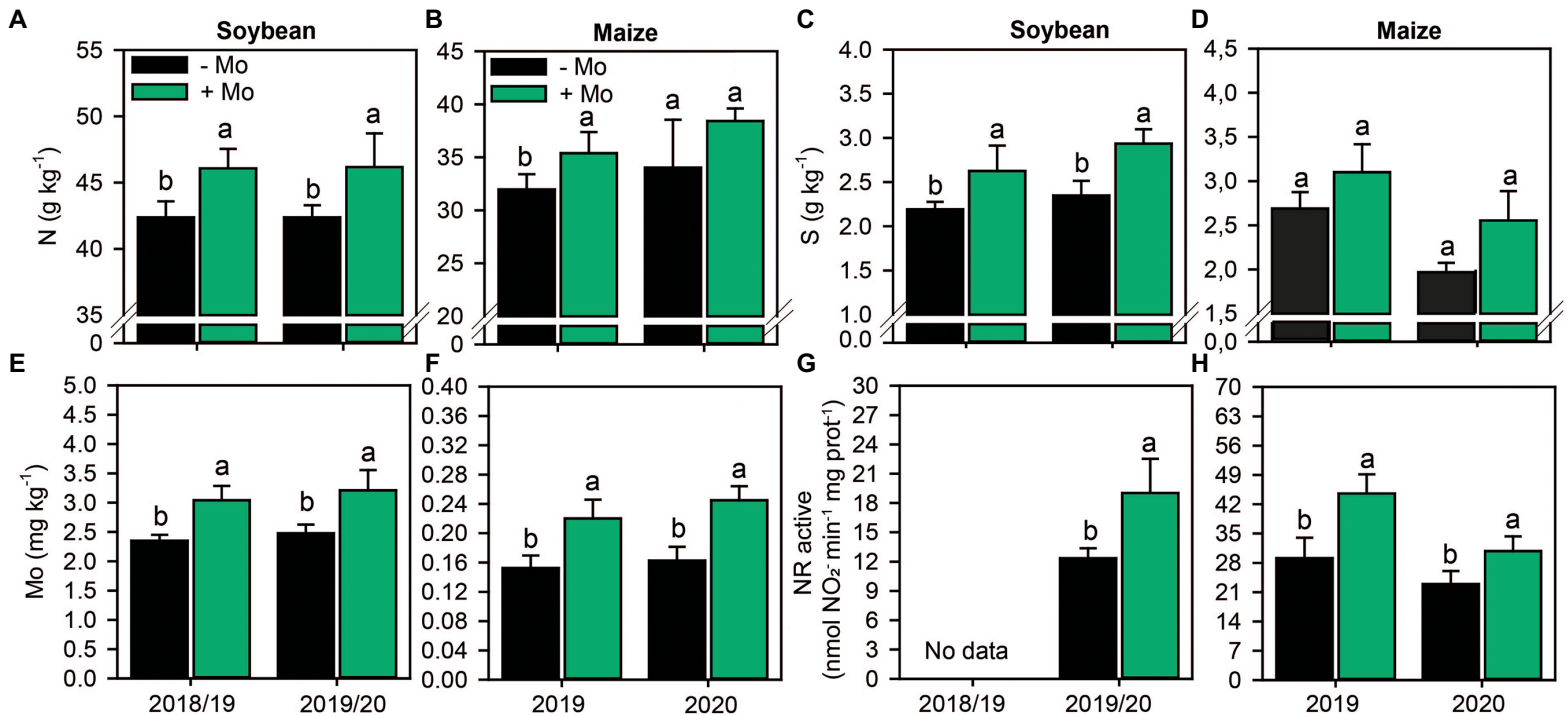
Nitrogen **(A,B)**, sulfur **(C,D)** and molybdenum **(E,F)** foliar content and nitrate reductase activity **(G,H)** in leaves of soybean and maize as affected by foliar Mo fertilization. Different lowercase letters represent statistical difference between treatments (presence or absence of Mo) according to the F test (*p* ≤ 0.10).

Foliar Mo fertilization improved foliar N content in both crops, with increases of 8 and 10% in soybean and maize, respectively (Figures 2A,B). In addition, foliar fertilization with Mo increased leaf S content by 16% and 20% in soybean in the first and second growing seasons, respectively, and by 23% in maize in the second growing season (Figures 2C,D). Other macronutrients were not impacted by Mo fertilization (*p* ≤ 0.10; Supplementary Figure S1).

Foliar spraying increased Mo content by 22% and 23% in soybean leaves and 32% and 34% in maize leaves in the first and second growing seasons, respectively (Figures 2E,F). Other micronutrients were not affected by Mo foliar spraying (*p* ≤ 0.10; Supplementary Figure S2). Consequently, application of Mo increased the active NR by 35% in the second soybean growing season and by 35% and 25% in the first and second maize growing seasons, respectively, compared with the control (Figures 2G,H). Foliar fertilization impacted total NR only in the second maize growing season, with an increase of 38% (Supplementary Figure S3).

### Photosynthetic Parameters and Carbon Assimilation

**Figure 3:**
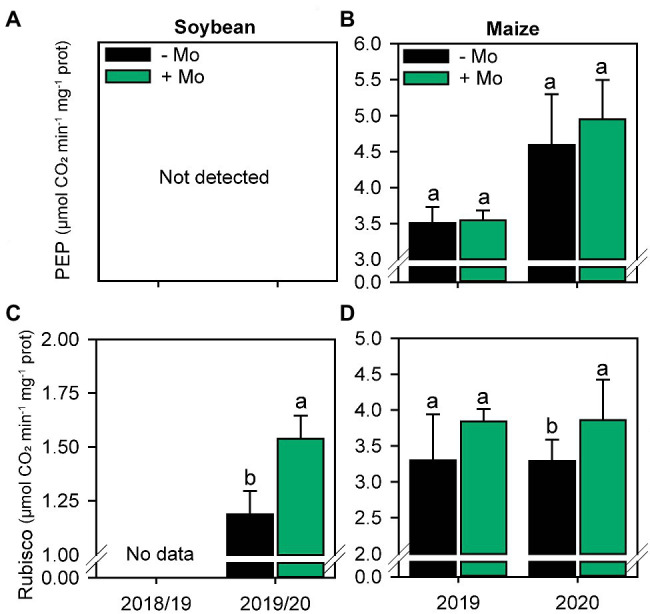
Activity of PEP Carboxylase **(A,B)** and Rubisco **(C,D)** enzymes of soybean and Maize plants under the effect of the presence or absence of foliar application of Mo. Different lowercase letters represent statistical difference between treatments (presence or absence of Mo) according to the F test (*p* ≤ 0.10).

The activity of the enzyme PEP carboxylase in maize was not altered by the application of Mo (Figure 3B). However, Rubisco activity increased by 8% and 14% in soybean and maize, respectively, in the second growing season (Figures 3C,D).

**Figure 4:**
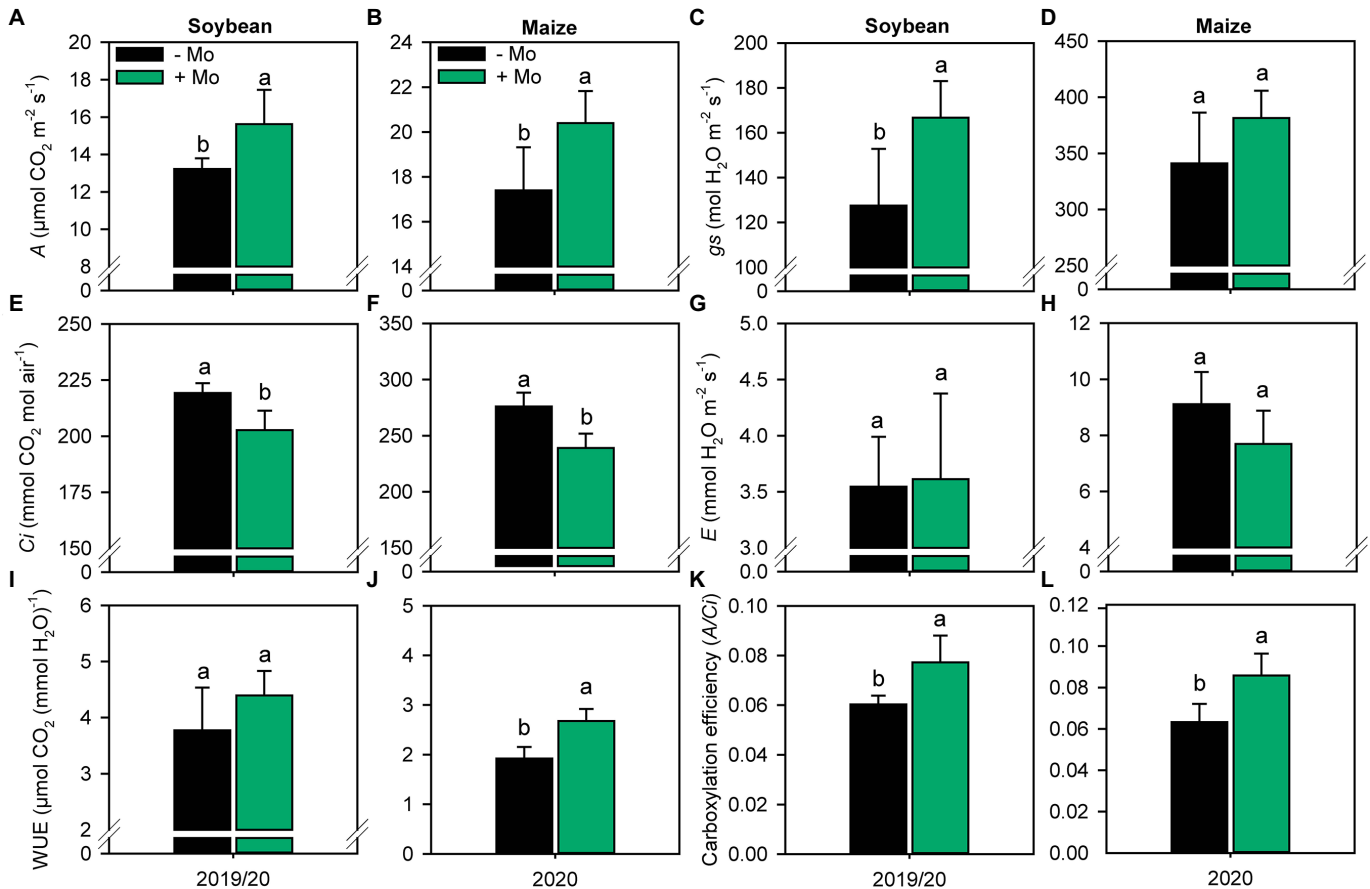
Net photosynthetic rate, *A* **(A,B)**; stomatal conductance, *gs* **(C,D)**; substomatal CO_2_ concentration, *Ci* **(E,F)**; leaf transpiration, *E* **(G,H)**; water use efficiency, WUE **(I,J)**; and carboxylation efficiency, *A/Ci* **(K,L)** of soybean and maize plants as affected by foliar Mo fertilization. Different lowercase letters represent statistical difference between treatments (presence or absence of Mo) according to the F test (*p* ≤ 0.10).

Foliar application of Mo improved photosynthetic activity compared with the control, with increases in net photosynthesis (*A*; Figures 4A,B) of 15% and 14% for soybean and maize, respectively. The stomatal conductance (*gs*) increased only for soybean by 23% (Figure 4C). The internal CO_2_ concentration (*Ci*) decreased by 8% in soybean and 13% in maize (Figures 4E,F), resulting in increases in photosynthetic efficiency (*A/Ci*) of 21 and 26% (Figures 4K,L), respectively. Foliar spraying also improved WUE by 28% in maize (Figure 4J).

**Figure 5:**
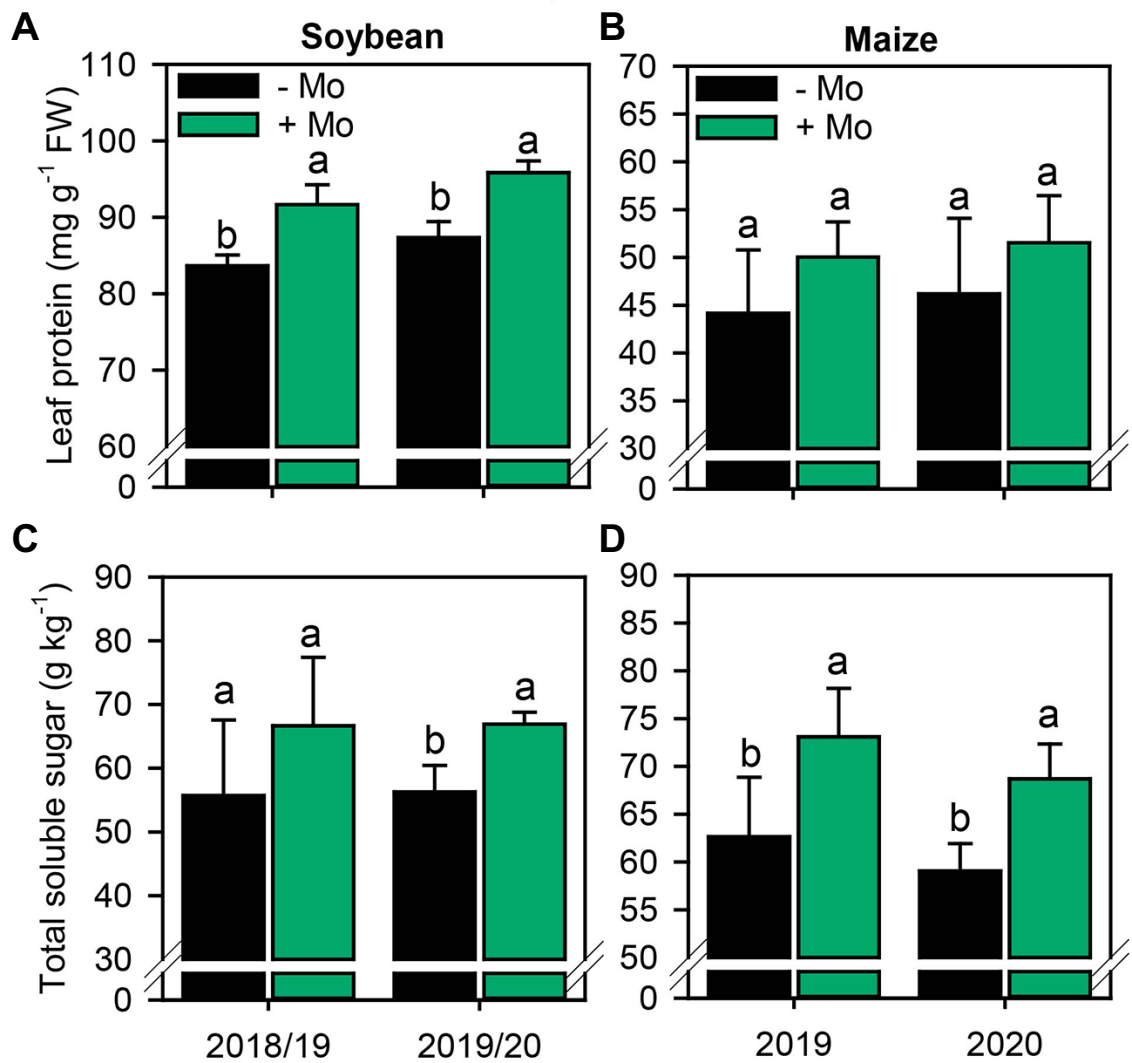
Leaf protein and total sugar concentration in soybean **(A,C)** and maize **(B,D)** leaves as affected by foliar Mo fertilization. Different lowercase letters represent statistical difference between treatments (presence or absence of Mo) according to the F test (*p* ≤ 0.10).

In both soybean growing seasons, Mo application increased leaf protein content by 9% (Figure 5A). By contrast, there was no significant response in maize (*p* ≤ 0.10; Figure 5B). Mo spraying increased total soluble sugar content by 16%, in second growing season of soybean, and 14% in maize in both growing seasons (Figures 5C,D).

### Shot Dry Matter and Shoot N Accumulation

**Figure 6:**
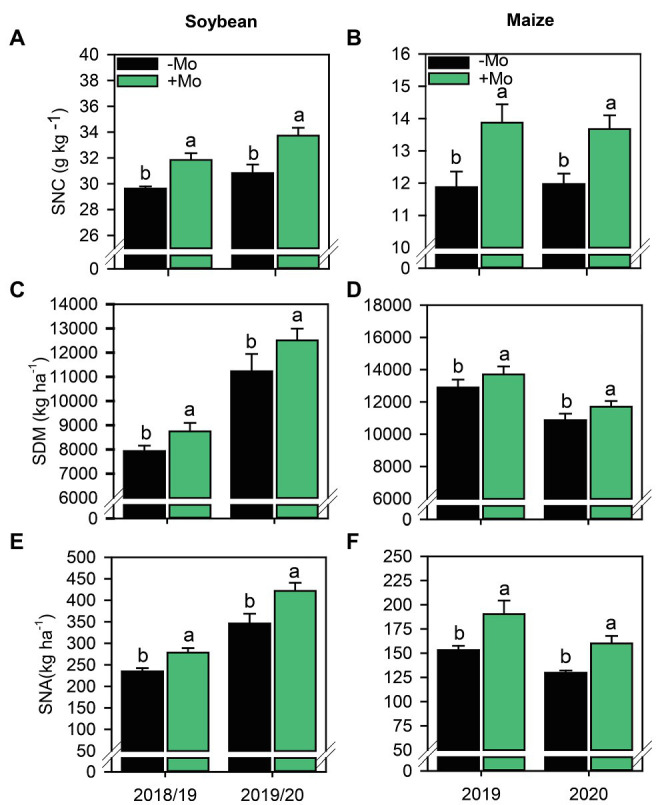
Shoot nitrogen concentration **(A,B)**, Shoot dry matter **(C,D)** and Shoot nitrogen accumulated **(E,F)** of soybean and maize plants as affected by presence or absence of the foliar Mo application. Different lower-case letters indicate significant differences between treatments (presence or absence of Mo supplementation) according to the F test (*p* ≤ 0.10). Growing seasons was considered as random effects.

Mo foliar fertilization increased the SNC in the plant by 7.0% and 8.6% in soybean (Figure 6A), and 14.3% and 12.6% in maize (Figure 6B), referring to the first and second growing season, respectively. SDM in soybean and maize in both growing seasons also increased with the application of Mo. The percentage of increase for soybeans corresponded to 9.3% and 10.2% and for maize, the increases were 5.9% and 7.0%, corresponding to the first and second growing seasons, respectively (Figures 6C,D). The SNA was also affected by fertilization with Mo in both crops, with increases of 15.6% and 17.9% in soybean, and 19.5% and 18.9% in maize, referring to the first and second growing seasons, respectively (Figures 6E,F).

### Yield Components and Grain Yield

**Figure 7:**
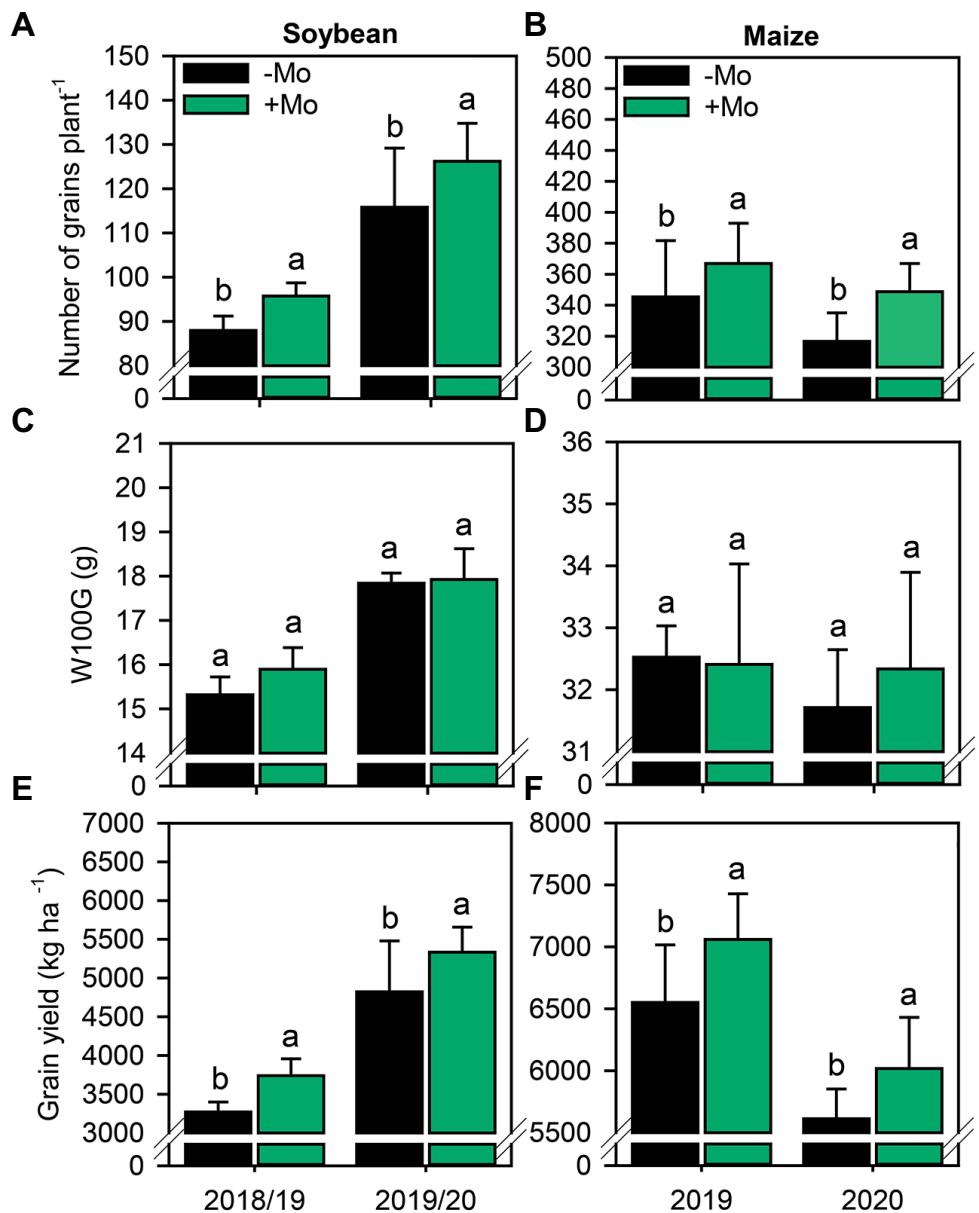
Number of grains per plant **(A,B)**, mass of 100 grains **(C,D)** and grain yield **(E,F)** in soybean and maize plants as affected by foliar Mo fertilization. Different lowercase letters represent statistical difference between treatments (presence or absence of Mo) according to the F test (*p* ≤ 0.10).

Overall, Mo spraying increased the yield components of soybean and maize. The number of pods per soybean plant increased by 9.6% and 7.2% in the first and second growing seasons, respectively (Supplementary Figure S4A). By contrast, the prolificacy of maize plants was not impacted by Mo application (*p* ≤ 0.10; Supplementary Figure S4C). Applying Mo did not significantly influence the number of grains per soybean pod (Supplementary Figure S4B) but increased the number of grains per maize ear by ~6% in both growing seasons (Supplementary Figure S4D). Taken together, these changes impacted the NGP, which increased by 5.9% and 8.5% in maize and 8.1% and 12.2% in soybean in the first and second growing seasons, respectively (Figures 7A,B). However, nutritional management had no impact on W100G (*p* ≤ 0.10; Figures 7C,D). Finally, the GY of both crops was improved by spraying with Mo (Figures 7E,F). In the first soybean growing season, the yield was 3,270 kg ha^−1^ and 3,740 kg ha^−1^ in the control and Mo application treatments, respectively. In the second growing season, the control treatment reached a productivity of 4,820 kg ha^−1^, while the treatment with Mo application reached 5,350 kg ha^−1^, an increase of approximately 10%. In the first maize growing season, the yield was 6,550 kg ha^−1^ in the control but 7,060 kg ha^−1^ under Mo application, an increase of 7.2%. In the second maize growing season, the yields were 5,610 kg ha^−1^ and 6,140 kg ha^−1^ in the control and Mo application treatments, respectively, corresponding to a yield increase due to Mo of ~8.6%. On average, soybean and maize yields increased by 500 kg ha^−1^ and 520 kg ha^−1^, respectively, under foliar Mo fertilization.

### Pearson’s Correlation Among Soybean and Maize Parameters

**Figure 8:**
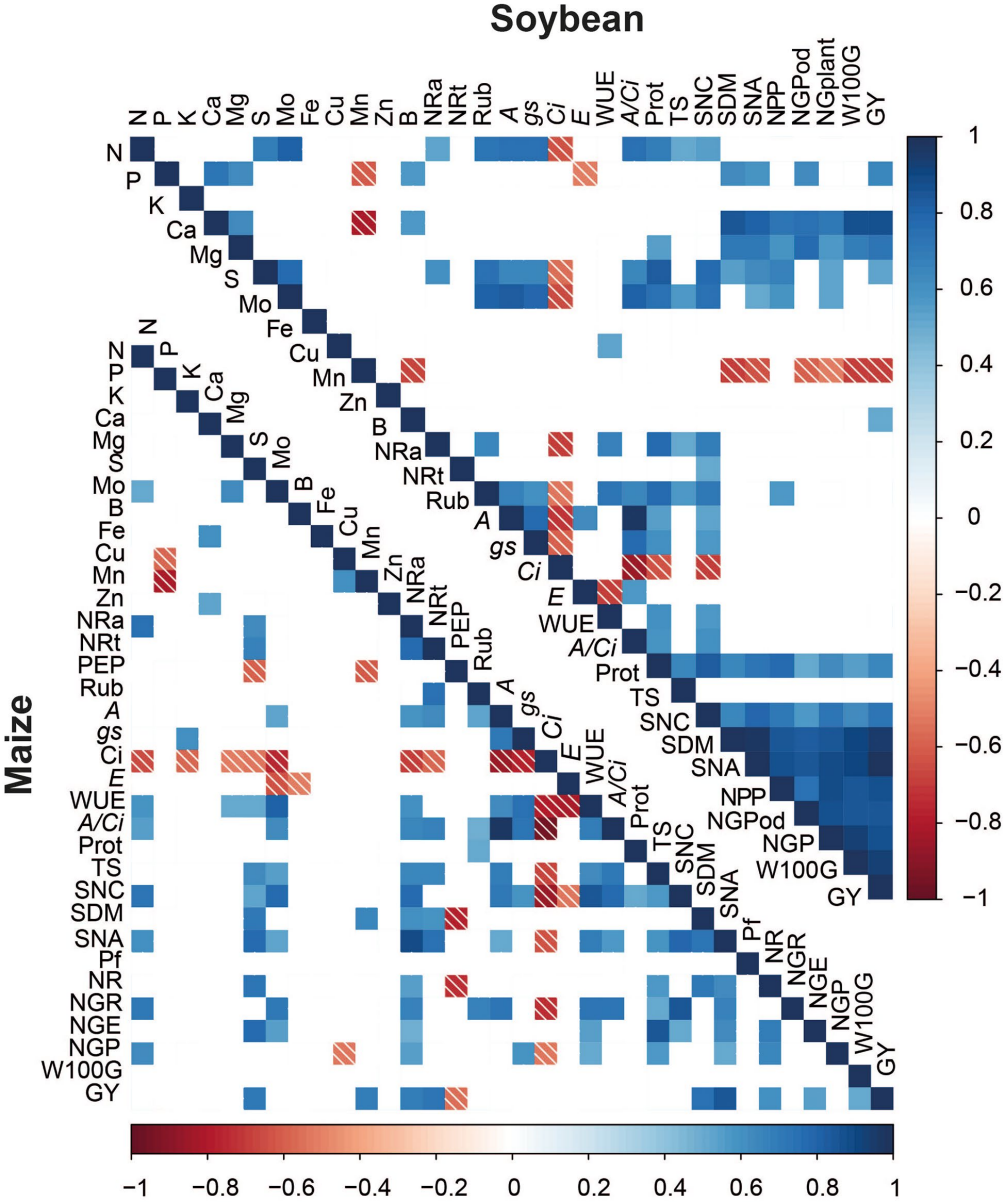
Heatmap of Pearson’s Correlation coefficients. Nitrogen (N), Phosphorus (P), Potassium (K), calcium (Ca), Magnesium (Mg), sulfur (S), Molybdenum (Mo), Boron (B), Iron (Fe), Copper (Cu), Manganese (Mn), Zinc (Zn), Nitrate reductase active (NRa), Total nitrate reductase (NRt), PEP carboxilase (PEP), Rubisco (Rub), Net photosynthesis rate (A), stomatal conductance (*gs*), internal CO_2_ concentration (*Ci*), leaf transpiration (*E*) water use efficiency (WUE), carboxylation efficiency (*A/Ci*), Protein (Prot), leaf total sugar concentration (TS), Shoot nitrogen concentration (SNC), Shoot dry matter (DM), Shoot nitrogen accumulation (NA), Prolificacy (Pf), number of rows per ear (NR), number of grains per rows (NGR), number of grains per ear (NGE), number of pods per plant (NPP), number of grains per plant (NGP), mass of 100 grains (W100G), and grain yield (GY).

A greater number of positive correlations was verified for the soybean crop, mainly between the production components SNC, SDM, SNA, NPP, NGP, W100G, and GY (Figure 8). In soybean, Mo correlated positively with the leaf contents of S, N, Rubisco, *A*, *gs*, *A/Ci*, Protein, TS, SNC, SNA, NPP, and NGP. In maize, Mo leaf content correlated with N leaf content, *A*, WUE, *A/Ci*, TS, SNC, SNA, NG and NGE. SNA correlated with GY in both crops, which may be one of the ways to increase crop yields.

## Discussion

Mo plays an important role in several plant metabolic processes, most notably biological nitrogen fixation and nitrate reduction (Calonego et al., 2010; Steiner and Zoz, 2015; Crusciol et al., 2019). Recent studies have reported direct effects of Mo on photosynthesis *via* metabolic pathways that are not yet fully understood (Yu et al., 2006; Imran et al., 2019; Rana et al., 2020). However, most of these works were performed under controlled conditions, and the implications for efficacy under field conditions are therefore not clear. In addition, few studies have examined the efficiency of foliar Mo fertilization in soybean-maize succession systems. The results of the present study show that foliar fertilization with Mo can modulate NR activity and processes involved in photosynthesis in soybean and maize, resulting in increased GY.

In general, foliar application of Mo improved the physiology and productivity of the studied crops. As expected, leaf N concentration was significantly increased in both crops, as Mo is directly involved in N metabolism and acts on important enzymes such as nitrogenase, xanthine dehydrogenase and NR, which are involved in N absorption, transport and conversion of NO_3_^−^ to NO_2_^−^, respectively (Bittner, 2014). In particular, the activity of NR, mainly its active form, increased, suggesting that the localized application of Mo to leaves effectively increased the expression and activation of NR, resulting in greater N assimilation by the crops. The absence of foliar Mo supplementation favored the accumulation of nitrate in leaves, indicating the low efficiency of the assimilation of nitrate under low concentrations of this micronutrient (Calonego et al., 2010). In leaves, the activation of NR is dependent on photosynthesis and is inactivated when light intensity is reduced or the leaf concentration of CO_2_ is low (Kaiser and Spill, 1991; Provan and Lillo, 1999). Therefore, increased photosynthesis may also contribute to an increase in the concentration of active NR. On the other hand, the reduction and assimilation of N will benefit the whole photosynthetic apparatus (enzyme production) and increase photosynthesis.

Applying Mo also improved S content by increasing N content. N interacts with S, and thus a deficiency of one element reduces the absorption and assimilation of the other. The absorption and assimilation of S depend on a constant supply of the cysteine precursor, O-acetylserine, which in turn depends on an adequate supply of N (Abdin et al., 2003).

The increased Mo content in soybean and maize leaves indicated that this nutrient was efficiently absorbed by leaves. Absorbed Mo can be translocated to other organs *via* the phloem to reach, for example, the nodules of soybean plants within 5 days of application (Campo and Hungria, 2002; Steiner and Zoz, 2015).

The range of Mo sufficiency in the leaf tissue is 1–5 mg kg^−1^ for soybean and 0.1–1 mg kg^−1^ for maize (Walsh and Beaton, 1975; Novais et al., 2007). In all treatments, Mo was sufficient for both crops, but supplementation with Mo improved photosynthetic activity by increasing net photosynthesis (*A*) and stomatal conductance (*gs*) and reducing *Ci*. These improvements, even in well-nourished plants, support the use of stimulant foliar fertilization for plants with high yield potential to correct nutrient composition and improve plant metabolism (Rosolem, 2002). In summary, foliar fertilization with Mo may maximize physiological processes of the plant such as photosynthesis, ultimately leading to increased GY.

Typically, *gs* is used to measure the capacity of stomatal opening and closing in response to environmental stimuli, whereas *Ci* and *A* are used to measure the efficiency of carbon fixation by plants (Farquhar and Sharkey, 1982). Stomata are responsible for the exchange of carbon and water between the interior of the leaf and the atmosphere, processes that are fundamental to photosynthesis (Gago et al., 2016). Thus, the greater the *gs*, the greater the diffusion of CO_2_ into the leaf, and this, combined with low levels of *Ci*, indicates that the mesophyll cells were able to assimilate CO_2_ retained in the substomatal chamber through photosynthesis (Lawson and Blatt, 2014).

The increase in photosynthetic activity mediated by Mo supplementation may be due to two main effects: indirect effects of Mo on leaf N supply due to the participation of Mo in the synthesis of proteins such as Rubisco and direct effects due to the roles of Mo in chlorophyll biosynthesis and the stability of the photosynthetic apparatus (Yu et al., 2006; Carmo-Silva et al., 2015; Steiner and Zoz, 2015; Imran et al., 2019). Several studies have confirmed that the application of Mo improves chlorophyll content (Bambara and Ndakidemi, 2009; Wu et al., 2017; Li et al., 2018; Liu et al., 2020; Rana et al., 2020). In addition, Mo spraying has been reported to increase the number of chloroplasts and improve the stability of the cell wall and plasma membrane of thylakoids (Weng et al., 2009). Mo may also be involved in cytochrome *b*_6_-*f* stability (Sun et al., 2014) and in improving chloroplast ultrastructure (Imran et al., 2019).

The improvement in photosynthetic activity was accompanied by increased WUE in maize, as leaf-level WUE reflects the ratio of net photosynthesis to transpiration (*A/E*). Since *E* was not affected by Mo application, carbon fixation increased under the same water use when Mo was applied (Figures 4I,J). During the time period of the study, significant dry spells occurred, especially in the second maize growing season (Figures 1B,D). The considerable increase in WUE in the presence of Mo compared with the control suggests that this element can improve tolerance to water deficit, ensuring an increase in photosynthetic and productive components even under water restriction. WUE is an important component of agricultural sustainability, especially in light of predictions of more frequent episodes of climatic anomalies such as droughts and heatwaves (Bhattacharya, 2019).

The increased protein content in soybean leaves may be the result of increased N and S content and photosynthetic activity. Moreover, increased N content is a strong indication that Mo application enhances N absorption and translocation. N is an essential structural element of proteins and accounts for 16%–23% of the content of plant proteins (Yeoh and Wee, 1994). In leaves, the most abundant protein is the enzyme Rubisco, which represents up to 50% of total soluble protein (Carmo-Silva et al., 2015). Increased protein content may therefore indicate an increase in Rubisco content. In support of this hypothesis, Maekawa and Kokubun (2005) observed a positive correlation between Rubisco content in soybean leaves and the applied dose of N. In addition, increased photosynthesis due to increased Rubisco levels has the potential to create a positive feedback effect by stimulating NR activation, which favors the assimilation of N and the onset of protein translation (Kaiser and Spill, 1991; Tcherkez et al., 2020).

Mo application did not alter PEPcase enzyme activity in maize leaves but did increase Rubisco enzyme activity in both crops. Mo fertilization has been reported to promote Rubisco stability under abiotic stress conditions (Sun et al., 2014). Together, the increased stability of Rubisco and higher protein content under Mo application could explain the improvement in the activity of this enzyme under drought stress. The increase in Rubisco enzyme activity led to an increase in total soluble sugar content, reflecting improved photosynthetic efficiency (Steiner and Zoz, 2015). Based on these results, it can be argued that Mo increases total soluble sugar by regulating photosynthesis. The high leaf soluble sugar content promoted by Mo fertilization may have been transported from the source to sinks *via* the phloem, resulting in increased NGP. Carbohydrate allocation differed between soybean and maize, as the number of pods per soybean plant increased, while maize did not change prolificacy. By contrast, the number of kernels per maize ear increased, whereas the number of kernels per soybean pod did not. Overall, grain yield increased by approximately 500 kg ha^−1^ for both crops. This cascade of events culminated in a greater production of SDM which, associated with an increase in SNC, led to greater SNA. These results show how the localized application of micronutrients in small concentrations can change the entire metabolic mechanism of the plant, reflecting greater biological production.

Several studies of Mo fertilization have attributed increased GY to improvements in biological nitrogen fixation and NR activity (Campo et al., 1999; Calonego et al., 2010; Mercante et al., 2011; Steiner and Zoz, 2015; Crusciol et al., 2019). In the present study, supplementation with Mo improved GY not only by increasing the absorption and assimilation of N but also by altering photosynthetic properties, despite conditions of water scarcity in the two growing seasons, especially for maize. This response is a strong indication that foliar-applied Mo may be an important strategy not only to supply nutrients but also to stimulate carbon metabolism.

In maize, drought stress during flowering and emission of stigma styles can greatly reduce the number of grains and grain yield (Magalhães and Durães, 2006). Here, Mo fertilization increased the number of grains and maize yield, in addition to WUE, despite drought stress during the reproductive stage in both growing seasons. Although these findings represent a great advance in the understanding of the nutritional effects and stimulant action of Mo, many gaps remain, especially in relation to its anti-stress role. Mo influences the synthesis of abscisic acid (ABA), a hormone responsible for stomatal control under stress, and may be involved in the modulation of antioxidant enzymes (Watanabe et al., 2018). However, the effects of Mo on ABA have not been tested under field conditions and will be explored in further studies to confirm the role of Mo in coping with climate adversity.

## Conclusion

In the present study, foliar Mo fertilization improved the physiology and productivity of soybean and maize crops by increasing NR activity, which in turn promoted increased leaf N content and protein synthesis. Foliar Mo fertilization also improved photosynthetic parameters, suggesting that foliar Mo fertilization is a viable strategy not only for promoting the nutritional status of the plant but also for stimulating carbon metabolism.

No adverse effects of Mo application were observed in the present study. Conversely, the positive effects of foliar fertilization with Mo were clear with the application of only 30 g Mo ha^−1^. Both soybean and maize commercial crops are sprayed several times during their cycles for other nutrients and agrochemicals. Mo may be included in one of these spray campaigns with little or no additional cost but with significant potential benefits. Therefore, it is suggested that foliar Mo fertilization be included in soybean and maize nutrient management.

## Data Availability Statement

The raw data supporting the conclusions of this article will be made available by the authors, without undue reservation.

## Author Contributions

CC, JC, and HC designed the experiment. VR and SO obtained and processed the data. JP, JB, and TG analyzed the data. SO, LM, JC, JP, JB, and TG wrote the paper with contribution of all authors. All authors confirm being contributor of this work and has approved it for publication.

## Funding

This study was financed by the National Council for Scientific and Technological Development (CNPq), providing the scholarship to the SO (grant number: 130646/2019-9).

## Conflict of Interest

The authors declare that the research was conducted in the absence of any commercial or financial relationships that could be construed as a potential conflict of interest.

## Publisher’s Note

All claims expressed in this article are solely those of the authors and do not necessarily represent those of their affiliated organizations, or those of the publisher, the editors and the reviewers. Any product that may be evaluated in this article, or claim that may be made by its manufacturer, is not guaranteed or endorsed by the publisher.
