# Supplementary material for: Molybdenum Foliar Fertilization Improves Photosynthetic Metabolism and Grain Yields of Field-Grown Soybean and Maize
Source: Front Plant Sci. 2022 May 25;13:887682. doi: 10.3389/fpls.2022.887682 (PMC9199428; doi:10.3389/fpls.2022.887682)
Supplement: Supplementary file 1 [file Data_Sheet_1.docx]

**Supplementary material**

**Table S1.** Physicochemical attributes (0.0–0.2-m depth) before sowing.

| **Soil Properties** | | **Unit** | **Value** |
| --- | --- | --- | --- |
| **Physical** |  |  |  |
| Clay |  | g kg-1 | 602 |
| Silt |  | g kg-1 | 281 |
| Sand |  | g kg-1 | 117 |
| Bulk density | | g cm−3 | 1.19 |
| **Chemical** | | | |
| pH _(CaCl2)_ |  | – | 5.4 |
| Soil organic matter | | g kg-1 | 22.0 |
| Phosphorus–available (P _resin_) | | mg kg−1 | 29.0 |
| Exchangeable | Calcium (Ca2+ resin) | mg kg−1 | 1162.3 |
|  | Magnesium (Mg2+ resin) | mg kg−1 | 182.3 |
|  | Potassium (K+ resin) | mg kg−1 | 109.5 |
|  | Aluminum (Al3+ KCl) | mg kg−1 | 18.0 |
| Potential acidity (H+Al) | | mg kg−1 | 50.4 |
| S-Sulfate (S–SO_4_2- Ca(H2PO4)2) | | mg kg−1 | 4.9 |
| Boron (B Hot water) | | mg kg−1 | 0.4 |
| Copper (Cu _DTPA-TEA_a) | | mg kg−1 | 8.8 |
| Iron (Fe DTPA-TEA) | | mg kg−1 | 22.0 |
| Manganese (Mn _DTPA-TEA_) | | mg kg−1 | 26.2 |
| Zinc (Zn DTPA-TEA) | | mg kg−1 | 2.1 |
| Molybdenum (Mo DTPA-TEA) | | mg kg−1 | 1.2 |
| Base saturation (BS) | | % | 62.0 |
| Cation exchange capacity (CEC _pH_ _7.0_) | | mmol_c_ kg−1 | 90.0 |

aDTPA-TEA_,_ diethylenetriaminepentaacetic acid-triethanolamine;


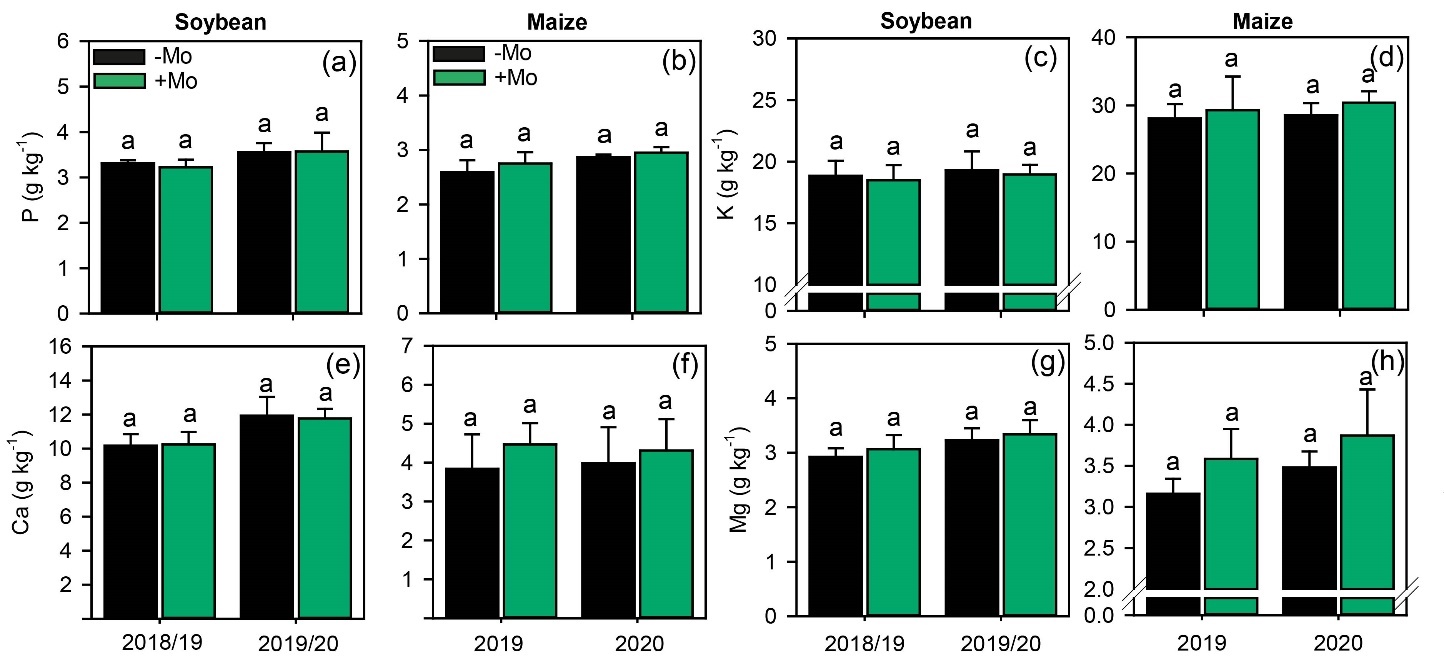


**Figure S1.** Macronutrient contents in soybean and maize leaves as affected by foliar Mo fertilization. P (A, B), K (C, D), Ca (E, F), Mg (G, H), Different lowercase letters represent statistical difference between treatments (presence or absence of Mo) according to the F test (*p*≤0.10).


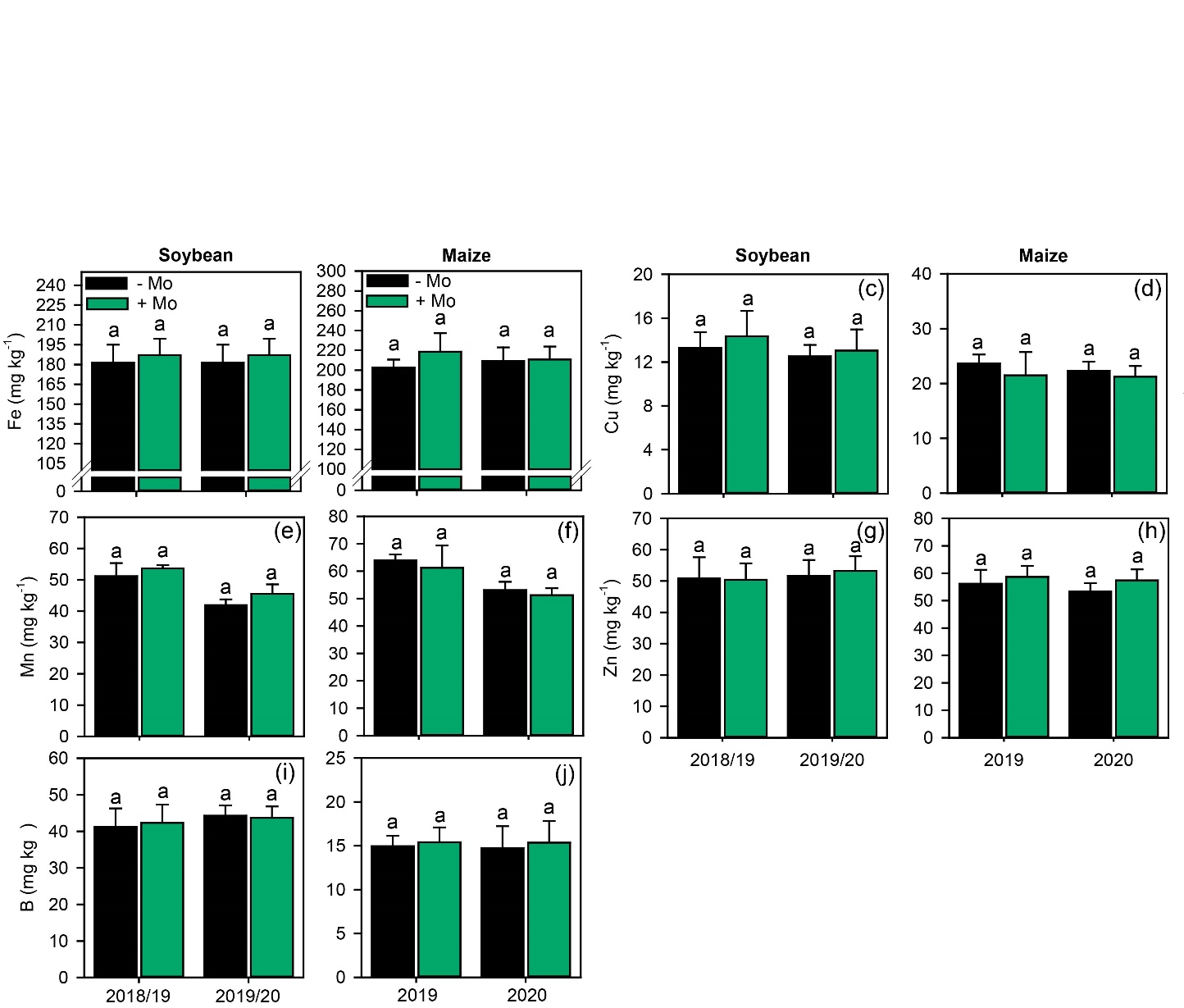


**Figure S2.** Micronutrient contents in soybean and maize leaves as affected by foliar Mo fertilization. Fe (A, B); Cu (C, D); Mn (E, F); Zn (G, H); B (I, J). Different lowercase letters represent statistical difference between treatments (presence or absence of Mo) according to the F test (*p*≤0.10).


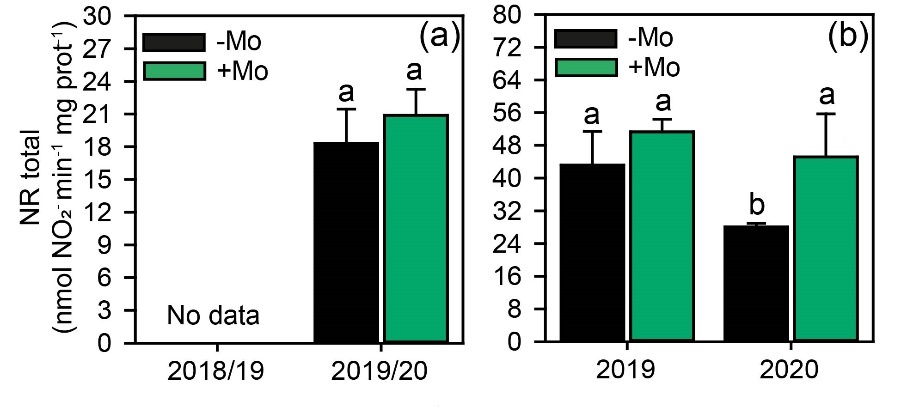


**Figure S3**. Total Nitrate reductase (A, B) in soybean and maize leaves as affected by foliar Mo fertilization. Different lowercase letters represent statistical difference between treatments (presence or absence of Mo) according to the F test (*p*≤0.10).


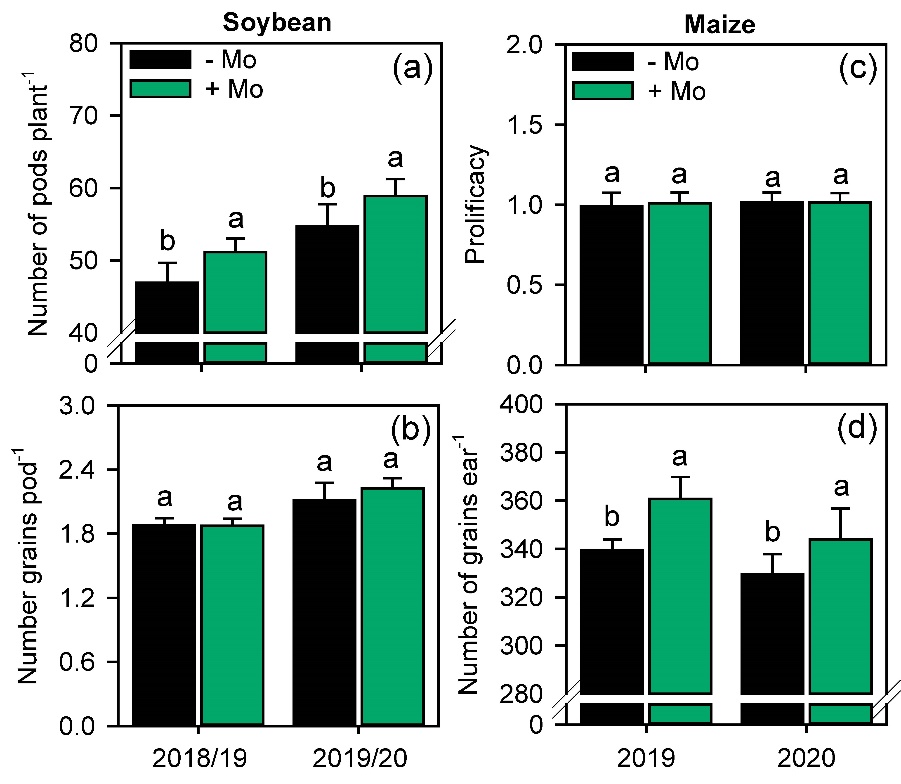


**Figure S4**. Number of pods per plant (A), number of grains per pod (B) in soybean plants and prolificacy (C), number of grains per ear (D) in soybean and maize plants as affected by foliar Mo fertilization. Different lowercase letters represent statistical difference between treatments (presence or absence of Mo) according to the F test (*p*≤0.10).
